# HJ-4, a novel piperine derivative, inhibits tumor growth and angiogenesis via p53 activation and oncogenic pathway inhibition in colorectal cancer models

**DOI:** 10.1038/s41598-025-18290-6

**Published:** 2025-09-29

**Authors:** Luyao Zhang, Shunfang Liu, Dan Wang, Xingyu Zhang, Zhongke Hu, Xun Zou, Xiuming Li, Xiujun Wang, Dandan Xu, Wei Liu, Bin Liu

**Affiliations:** 1 Jiangsu Key Laboratory of Marine Pharmaceutical Compound Screening, College of Pharmacy, Jiangsu Ocean University, Lianyungang, 222005 China; 2 Department of Oncology, Tongji Hospital of Tongji Medical College, Huazhong University of Science and Technology, Jiefang Road 1095, Wuhan, 430030 China; 3 Cancer Center and Department of Pharmacology and Toxicology, Medical College of Wisconsin, Milwaukee, WI 53226 USA

**Keywords:** Colorectal cancer (CRC), Piperine derivative, HJ-4, Angiogenesis inhibition, p53-mediated apoptosis, Chicken embryo chorioallantoic membrane (CAM) model, Biological techniques, Cancer, Drug discovery

## Abstract

Colorectal cancer (CRC) remains a major cause of cancer-related mortality worldwide, especially in advanced and metastatic stages where treatment options are limited. HJ-4, a novel piperine derivative, demonstrated strong tumor-selective inhibition. Within safe concentrations (cell viability > 85%), HJ-4 dose-dependently suppressed colony formation and DNA synthesis in CRC cells, showing potent anti-proliferative effects. It also significantly inhibited cell adhesion, wound healing, and invasion, indicating robust anti-migration and anti-invasion properties. In vivo, the CAM model confirmed that HJ-4 reduced both tumor volume and angiogenesis. Mechanistically, HJ-4 activated the p53-dependent apoptosis pathway while suppressing the Wnt/β-catenin axis and E2F transcriptional activity, effectively impeding tumor progression. Overall, HJ-4 exhibits promising tumor specificity and multiple antitumor mechanisms, supporting its potential for clinical development in CRC treatment.

**Supplementary Information:**

The online version contains supplementary material available at 10.1038/s41598-025-18290-6.

## Introduction

Colorectal cancer (CRC) is the third most commonly diagnosed malignancy and the second leading cause of cancer-related deaths worldwide, accounting for over 900,000 deaths annually^1,2^. Despite advances in diagnostic techniques and therapeutic strategies, the prognosis for patients with advanced or metastatic CRC remains poor, with a five-year survival rate typically below 15%^3^. Current treatment modalities—including surgery, chemotherapy, and targeted therapies—are limited by systemic toxicity, the emergence of drug resistance, and reduced efficacy against metastatic disease. These challenges underscore the urgent need for novel therapeutic agents with improved efficacy, specificity, and tolerability.

The initiation and progression of CRC involve multiple hallmarks of cancer, such as uncontrolled proliferation, evasion of apoptosis, angiogenesis, and metastasis^4^. Among these, the tumor suppressor protein p53 plays a pivotal role in maintaining genomic integrity by regulating cell cycle arrest, DNA repair, and apoptosis^5–7^. However, p53 is frequently mutated or inactivated in CRC, with approximately 50% of cases exhibiting aberrant p53 signaling, contributing to tumor development and treatment resistance^8^. Restoration or activation of p53 function has shown promise as a therapeutic strategy. In parallel, targeting oncogenic pathways such as Wnt/β-catenin^9,10^ and E2F signaling^11^ which drive CRC cell proliferation and metastasis, offers complementary approaches to inhibit tumor progression^12^. Given the complexity of CRC, agents capable of modulating multiple oncogenic pathways are particularly desirable.

Due to the limitations of conventional therapies, natural compounds have attracted significant attention in cancer prevention and treatment for their multi-targeted activities and low toxicity profiles^13^. Piperine, the major bioactive alkaloid from black pepper, has been reported to possess anti-inflammatory, antioxidant, and anti-cancer properties^14,15^. Studies have shown that piperine can inhibit CRC cell proliferation, induce apoptosis, suppress epithelial-mesenchymal transition (EMT), and modulate the gut microenvironment by regulating key pathways such as NF-κB^16^ Wnt/β-catenin, PI3K/Akt/mTOR^17^, and MAPK^18^. Moreover, piperine has been found to enhance the sensitivity of CRC cells to conventional chemotherapeutic agents, suggesting potential as an adjuvant therapy. However, its clinical utility is hampered by limitations such as low potency, poor selectivity, and unfavorable pharmacokinetic properties. Luckily, we discovered that HJ-4, a novel piperine derivative, exhibits superior anti-tumor efficacy compared to piperine in CRC models.

In this study, we report the synthesis and biological evaluation of HJ-4 in CRC, focusing on its ability to inhibit proliferation, migration, invasion, angiogenesis, and tumor growth. Mechanistic investigations were conducted to elucidate the molecular pathways regulated by HJ-4, with particular emphasis on its modulation of p53 signaling and apoptosis pathways. Our findings suggest that HJ-4 is a promising therapeutic candidate for CRC, demonstrating significantly improved potency and selectivity compared to piperine, and offering potential solutions to current challenges in CRC treatment.

## Materials and methods

### General materials and instruments

All reagents and solvents were purchased from commercial suppliers and used as received unless otherwise specified. Analytical-grade solvents were used for synthesis and purification. Thin-layer chromatography (TLC) was performed on silica gel 60 F-254 plates (Merck, Germany) and visualized under UV light (254 nm) or iodine vapor to monitor reactions. Column chromatography was conducted using silica gel (200–300 mesh or 300–400 mesh, Qingdao Marine Chemical Factory, China). The melting point of HJ-4 was determined using an X4 melting point tester (Shanghai Precision Scientific Instrument Co., China).

NMR spectra were acquired using Bruker Avance DRX spectrometers at 500 and 126 MHz with CDCl₃ as the solvent, and tetramethylsilane (TMS) as the internal standard. Chemical shifts (δ) are reported in parts per million (ppm), and coupling constants (*J*) are expressed in Hertz (Hz). High-resolution mass spectrometry (HRMS) was performed using a Thermo Scientific Orbitrap Elite spectrometer. The purity was analyzed by Shimadzu LC20AD high performance liquid chromatography (HPLC) coupled with a diode array detector (DAD) and the Inertsil ODS-3 column (EC-C18, 4.6 mm × 250 mm, 5 μm) with the eluent 77% CH_3_OH in pure water. The analysis process lasted for 20 min with a flow rate of 1.0 mL/min. RNA sequencing libraries were prepared and sequenced using an Illumina NovaSeq platform.

### Synthesis of HJ-4

#### Step 1: hydrolysis of Piperine to intermediate (2)

Piperine (5.85 g, 20.5 mmol) was dissolved in 20 mL of 20% potassium hydroxide (KOH) in ethanol and refluxed at 80 °C for 6 h with constant stirring under nitrogen atmosphere. The reaction mixture was cooled to room temperature, and the solid product was collected by filtration. The solid was dissolved in water, washed with methylene chloride (3 × 20 mL) to remove impurities, and the remaining aqueous phase was gradually acidified with 0.5 M hydrochloric acid (HCl) under stirring until the white precipitate formed. The precipitate was collected by vacuum filtration, washed with cold ethanol, and recrystallized to yield compound (**2**) as a white solid (3.51 g, yield: 78.5%).

#### Step 2: coupling of intermediate (2) with 1-(2-Methoxyphenyl)piperazine

Intermediate (**2**) (0.20 g, 0.916 mmol), EDCI·HCl (0.21 g, 1.1 mmol), HOBt (0.15 g, 1.1 mmol), and triethylamine (0.37 mL, 2.7 mmol) were dissolved in 4 mL of anhydrous dichloromethane under nitrogen. The mixture was stirred for 1 h at room temperature. Subsequently, 1-(2-methoxyphenyl)piperazine (0.21 g, 1.1 mmol) was added dropwise, and the reaction was continued for 2 h. Reaction progress was monitored by TLC. After completion, the solvent was evaporated under reduced pressure, and the crude product was purified by silica gel column chromatography using petroleum ether/ethyl acetate (3:1) as the eluent. HJ-4 was obtained as a white solid (0.21 g, yield: 58.4%). m.p.186.3 ~ 191.5 °C^1^. H NMR (500 MHz, CDCl_3_), δ 7.46 (dd, *J* = 14.6, 9.9 Hz, 1 H), 7.07–7.00 (m, 1 H), 6.99 (d, *J* = 1.6 Hz, 1 H), 6.96–6.86 (m, 4 H), 6.80–6.71 (m, 3 H), 6.45 (d, *J* = 14.6 Hz, 1 H), 5.97 (s, 2 H), 3.89–3.77 (m, 7 H), 3.10–3.04 (m, 4 H) (Fig. S1)^13^. C NMR (126 MHz, CDCl_3_), δ 165.7, 152.4, 148.4, 148.3, 143.2, 140.8, 138.9, 131.0, 125.3, 123.7, 122.7, 121.2, 119.5, 118.6, 111.5, 108.6, 105.8, 101.4, 55.6, 51.3, 50.7, 46.2, 42.4 (Fig. S2). HRMS *m/z*: [M + H]^+^ calcd for C_23_H_25_N_2_O_4_: 393.1809, found 393.1817 (Fig. S3). HPLC purity: 97.5% (Fig. S4).

### Cell lines and culture conditions

Human colorectal cancer cell lines HCT116 and SW480, cervical cancer cell line HeLa, kidney cancer cell line A498, and human renal epithelial cell line 293T were obtained from the American Type Culture Collection (ATCC). All cell lines were authenticated by short tandem repeat (STR) profiling and routinely tested for mycoplasma contamination using a MycoAlert Mycoplasma Detection Kit (Lonza, USA) before use. HCT116 cells were cultured in McCoy’s 5 A medium supplemented with 10% fetal bovine serum (FBS) and 1% penicillin/streptomycin. SW480 cells were maintained in Iscove’s Modified Dulbecco’s Medium (IMDM) supplemented with 10% FBS and 1% penicillin/streptomycin. HeLa and 293T cells were cultured in high-glucose Dulbecco’s Modified Eagle Medium (DMEM) supplemented with 10% FBS and 1% penicillin/streptomycin. A498 cells were grown in Minimum Essential Medium (MEM) containing 10% FBS and 1% penicillin/streptomycin. All cells were maintained in a humidified atmosphere of 5% CO₂ at 37 °C. Cells were passaged every 2–3 days using 0.25% trypsin-EDTA (Gibco, USA) and used within 10 passages for all experiments to ensure reproducibility.

### Cell viability assay

HCT116, SW480, A498, HeLa, and 293T cells were seeded into a 96-well plate at a density of 5 × 10³ cells per well. After allowing them to adhere overnight in a 37 °C, 5% CO₂ incubator, the cells were treated with HJ-4 or piperine at concentrations ranging from 3.125 to 100 µM for 24 h. After treatment, 10 µL of MTT solution (5 mg/mL) was added to each well, and the cells were incubated for an additional 4 h at 37 °C to allow for the formation of formazan crystals. Following incubation, the supernatant was carefully removed, and 100 µL of DMSO was added to dissolve the purple crystals. The absorbance at 490 nm was measured using a microplate reader to determine the optical density (OD) of each well. The relative cell viability was calculated by comparing the OD values between the experimental and control groups. The experiment included three replicate wells for each condition, along with untreated controls and blank wells to ensure the reliability of the data.

### Colony formation assay

HCT116 and SW480 cells were seeded in 12-well plates at a density of 500 cells per well and treated with HJ-4 (8, 16, and 32 µM) or piperine (32 µM) for 48 h. After treatment, the medium was replaced with drug-free complete medium, and cells were allowed to grow for 10–14 days. Colonies were fixed with 4% paraformaldehyde for 15 min, stained with 0.1% crystal violet for 30 min, and imaged using a digital scanner. Colonies containing more than 50 cells were manually counted using ImageJ software, and the average colony area was also analyzed.

### EdU incorporation assay

HCT116 and SW480 cells were seeded on coverslips in 24-well plates and treated with HJ-4 or piperine for 24 h. Cells were incubated with 5 µM EdU (Click-iT EdU Imaging Kit, Thermo Fisher Scientific) for 2 h, then fixed in 4% paraformaldehyde and permeabilized with 0.1% Triton X-100. The Click reaction was performed according to the manufacturer’s protocol, and nuclei were counterstained with Hoechst 33,342. Images were captured using a Zeiss LSM 880 confocal microscope, and the percentage of EdU-positive cells was quantified using ImageJ software.

### Adhesion assays

96-well plates were coated with 10 µg/mL human plasma fibronectin in PBS for 2 h at room temperature. After removing the coating solution, wells were blocked with 200 µL PBS containing 1% heat-denatured BSA at 37 °C for 1 h. HCT116 and SW480 cells were resuspended in serum-free medium with HJ-4 or piperine and seeded at 5 × 10^4^ cells/well. After cell attachment, wells were washed with PBS to remove non-adherent cells. MTT solution (100 µL, 0.5 mg/mL) was added and incubated at 37 °C for 4 h. The supernatant was discarded, and 100 µL DMSO was added to dissolve formazan crystals. Absorbance was measured at 490 nm. For parallel analysis, cells were fixed with 4% paraformaldehyde and imaged under a bright-field inverted microscope (10×). Cell coverage was quantified using ImageJ.

### Migration assays

HCT116/SW480 cells were seeded in 6-well plates at a density of 5 × 10⁵ cells per well. After the cells reached approximately 90% confluence, a linear wound was created by vertically drawing a line with a sterile 10 µL pipette tip approximately 800 μm wide along a ruler, perpendicular to the bottom of the plate. After washing with PBS to remove detached cells, the medium was replaced with serum-free medium containing different concentrations of drugs (HJ-4/Piperine). The plate was placed under an inverted fluorescence microscope, and images were captured at 0, 24, and 48 h using a 10× objective lens (3 random fields per well). Image analysis was performed using ImageJ: the images were first converted to 8-bit grayscale, and a fixed threshold (default settings) was applied to identify the wound edges.

### Invasion assays

The Transwell chamber’s base membrane was pre-coated with Matrigel matrix gel, diluted at a 1:8 ratio with pre-chilled serum-free medium, and 100 µL of this solution was added per well and allowed to solidify at 37 °C for 4 h. Drug-pretreated HCT116/SW480 cells (5 × 10^4^ cells in 200 µL serum-free medium) were seeded into the upper chamber, and the lower chamber was filled with 600 µL complete medium containing 10% FBS to serve as the chemotactic induction source. After 24 h of incubation, non-invasive cells in the upper chamber were removed with a cotton swab. The polycarbonate membrane was fixed with 4% paraformaldehyde for 20 min and stained with crystal violet for 15 min. Three random fields (10 × magnification) were selected under an inverted microscope, and the number of invasive cells was automatically counted using ImageJ. The final result was expressed as the percentage of invasion relative to the control group.

### RNA sequencing and transcriptomic analysis

HCT116 cells were treated with HJ-4 (32 µM) or DMSO (control) for 24 h in biological triplicates. Total RNA was extracted using the RNeasy Mini Kit (Qiagen, Germany) according to the manufacturer’s protocol. RNA quality and concentration were assessed using a NanoDrop 2000 spectrophotometer (Thermo Fisher Scientific, USA) and an Agilent 2100 Bioanalyzer (Agilent Technologies, USA). Samples with an RNA integrity number (RIN) ≥ 8.0 were used for library preparation. RNA sequencing libraries were constructed using the TruSeq RNA Sample Preparation Kit v2 (Illumina, USA). Libraries were sequenced on an Illumina NovaSeq 6000 platform to generate 150 bp paired-end reads. Raw sequencing reads were quality-checked using FastQC (v0.11.9) and trimmed with Trimmomatic (v0.39) to remove low-quality bases and adapters. Reads were aligned to the human reference genome (GRCh38) using STAR (v2.7.9a). FeatureCounts (v2.0.1) was used to quantify gene expression levels, and normalization was performed using the transcripts per million (TPM) method. Differentially expressed genes (DEGs) between HJ-4-treated and control groups were identified using the “limma” package in R (v4.1.0) with thresholds of |log₂ fold change| > 1.2 and adjusted *p*-value < 0.05. Gene Ontology (GO) enrichment and Kyoto Encyclopedia of Genes and Genomes (KEGG) pathway analyses were conducted using the “clusterProfiler” package. Gene set enrichment analysis (GSEA) was performed using the Molecular Signatures Database (MSigDB, v7.5). Pathway visualization was generated using the “enrichplot” and “ggplot2” packages in R. Volcano plots and heatmaps of DEGs were created using “EnhancedVolcano” and “pheatmap” packages, respectively.

### Western blot analysis

HCT116 and SW480 cells were treated with HJ-4 (8, 16, and 32 µM) or piperine (32 µM) for 24 h. After treatment, cells were washed twice with ice-cold phosphate-buffered saline (PBS) and lysed on ice in RIPA buffer (Thermo Fisher Scientific, USA) supplemented with protease inhibitors (Roche, Switzerland) and phosphatase inhibitors (Sigma-Aldrich, USA). Cell lysates were centrifuged at 12,000 × g for 15 min at 4 °C, and the supernatant was collected. Protein concentrations were determined using the BCA Protein Assay Kit (Thermo Fisher Scientific, USA) according to the manufacturer’s protocol. Equal amounts of protein (30–50 µg) were loaded onto 10–12% SDS-polyacrylamide gels, separated by electrophoresis, and transferred to polyvinylidene fluoride (PVDF) membranes (Millipore, USA) using a Bio-Rad wet transfer system at 100 V for 90 min. Membranes were blocked with 5% non-fat milk in TBS-T buffer (Tris-buffered saline containing 0.1% Tween-20) for 1 h at room temperature and incubated overnight at 4 °C with primary antibodies diluted in blocking buffer. The following primary antibodies were used: p53 (Cell Signaling Technology, CST 2524T, 1:1000), BAX (Proteintech, 60267-1-Ig, 1:1000), BCL-2 (Proteintech, 68103-1-Ig, 1:1000), PARP (Santa Cruz Biotechnology, sc-8007, 1:1000), cleaved PARP (Santa Cruz Biotechnology, sc-56196, 1:1000), caspase-3 (Beyotime, AF1213, 1:1000), cleaved caspase-3 (Beyotime, AF1261, 1:1000), and β-actin (Cell Signaling Technology, CST 4970, 1:2000), which served as the loading control. After washing three times with TBS-T, membranes were incubated with HRP-conjugated secondary antibodies (anti-mouse or anti-rabbit, CST 7076 S/7074S, 1:5000) for 1 h at room temperature, followed by three washes with TBS-T. Proteins were detected using an enhanced chemiluminescence (ECL) substrate (Thermo Fisher Scientific, USA), and images were captured using a Bio-Rad ChemiDoc XRS + Imaging System. Band intensities were quantified using ImageJ software, normalized to β-actin, and expressed as fold changes relative to the control group.

### Immunofluorescence assays

After HCT116 and SW480 cells were seeded onto glass coverslips, they were treated with HJ-4 (8, 16, 32 µM) and piperine (32 µM) for 24 h, respectively. After treatment, the cells were fixed with 4% paraformaldehyde at room temperature for 15 min, permeabilized with 0.1% Triton X-100 for 10 min to enhance membrane permeability, and then blocked with 5% BSA for 1 h to prevent nonspecific binding. The cells were incubated overnight at 4 °C with primary antibodies against p53 and BAX. Following this, corresponding species-specific Alexa Fluor secondary antibodies were applied and incubated at room temperature for 1 h in the dark. Finally, the cell nuclei were stained with DAPI for 5 min. After mounting with anti-fade mounting medium, samples were analyzed using a fluorescence microscope with multiple channels for image acquisition. Image overlay analysis was performed using ImageJ software. The untreated group was set as a negative control, and the experiment was repeated three times to verify the reliability of the results.

### YO-PRO-1/PI staining

HCT116 cells were seeded onto sterile cover slips placed in a 6-well plate and incubated overnight at 37 °C in a 5% CO₂ incubator to allow cell attachment. After attachment, different concentrations of HJ-4 (e.g., 8, 16, 32 µM) were added for 24 h. Following treatment, the culture medium was discarded, and cells were gently washed three times with pre-warmed PBS to remove any residual drug. After PBS removal, 1 µM YO-PRO-1 and 5 µg/mL propidium iodide (PI) were added as a mixed staining solution to each well, and cells were stained for 30 min at 37 °C in the dark. After staining, cells were washed again with PBS to remove excess dye, and the cover slips were inverted onto slides. Images were captured using a fluorescence microscope equipped with dual-channel filters, and the images were analyzed using ImageJ software to calculate the apoptosis/necrosis ratio for each treatment group.

### Chicken embryo chorioallantoic membrane (CAM) model

This study utilized the chicken embryo chorioallantoic membrane (CAM) model to evaluate the anti-tumor and anti-angiogenic effects of HJ-4. Fertilized eggs were obtained from Wufeng Poultry Farm in Tongling City, Anhui Province, China, and incubated at 37 °C with 50% humidity until the seventh day of embryonic development (E7). The eggs were opened at the air sac end (approximately 1.5 cm in diameter) using sterile techniques to expose the CAM. Tumor spheroids derived from HCT116 and SW480 colon cancer cell lines (prepared by adding 1 × 10⁶ cells to 50 µL of matrigel) were implanted into the vascular-rich regions of the CAM. Subsequently, HJ-4 (8, 16, 32 µM) or the positive control piperine (32 µM) was applied locally to the tumor site daily for 7 days. Tumor tissues were excised, weighed using an analytical balance, and morphological characteristics were recorded. Additionally, the number of tertiary blood vessels radiating in a 2 mm radius around the tumor was quantified to assess the anti-angiogenic effect. All procedures strictly followed the guidelines of the Biological Ethics Committee of Jiangsu Ocean University, and the egg windows were kept sealed throughout the experiment to ensure embryo survival. Each group consisted of six biological replicates to ensure data reliability.

### Statistical analysis

Data are presented as mean ± standard deviation (SD) from at least three independent experiments. Statistical comparisons between groups were performed using one-way analysis of variance (ANOVA) followed by Tukey’s post hoc test for multiple comparisons or Student’s t-test for two-group comparisons. *p*-values < 0.05 were considered statistically significant. All analyses were performed using GraphPad Prism 9.0 and R software.

## Results

### Synthesis of HJ-4

**Fig. 1:**
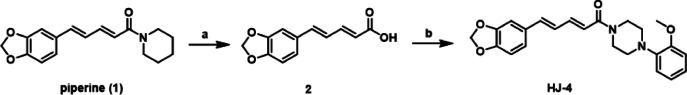
Synthetic approach to HJ-4. Reagents and conditions: (**a**) KOH, EtOH, reflux; (**b**) 1-(2-methoxyphenyl)piperazine, 3-(((ethylimino)methylene)amino)-*N*,*N*-dimethylpropan-1-amine hydrochloride (EDCI·HCl), 1-hydroxybenzotriazole (HOBt), Et_3_N, dichloromethane, rt.

Synthetic approach to HJ-4 is concise and described in Fig. 1. Initially, hydrolysis of the known natural product piperine with KOH afforded the corresponding carboxylic acid intermediate (**2**). Then EDCI-HOBt mediated condensation of **2** with the commercially available amine 1-(2-methoxyphenyl)piperazine successfully furnished the new amide HJ-4.

### In vitro antitumor activity of HJ-4

**Fig. 2:**
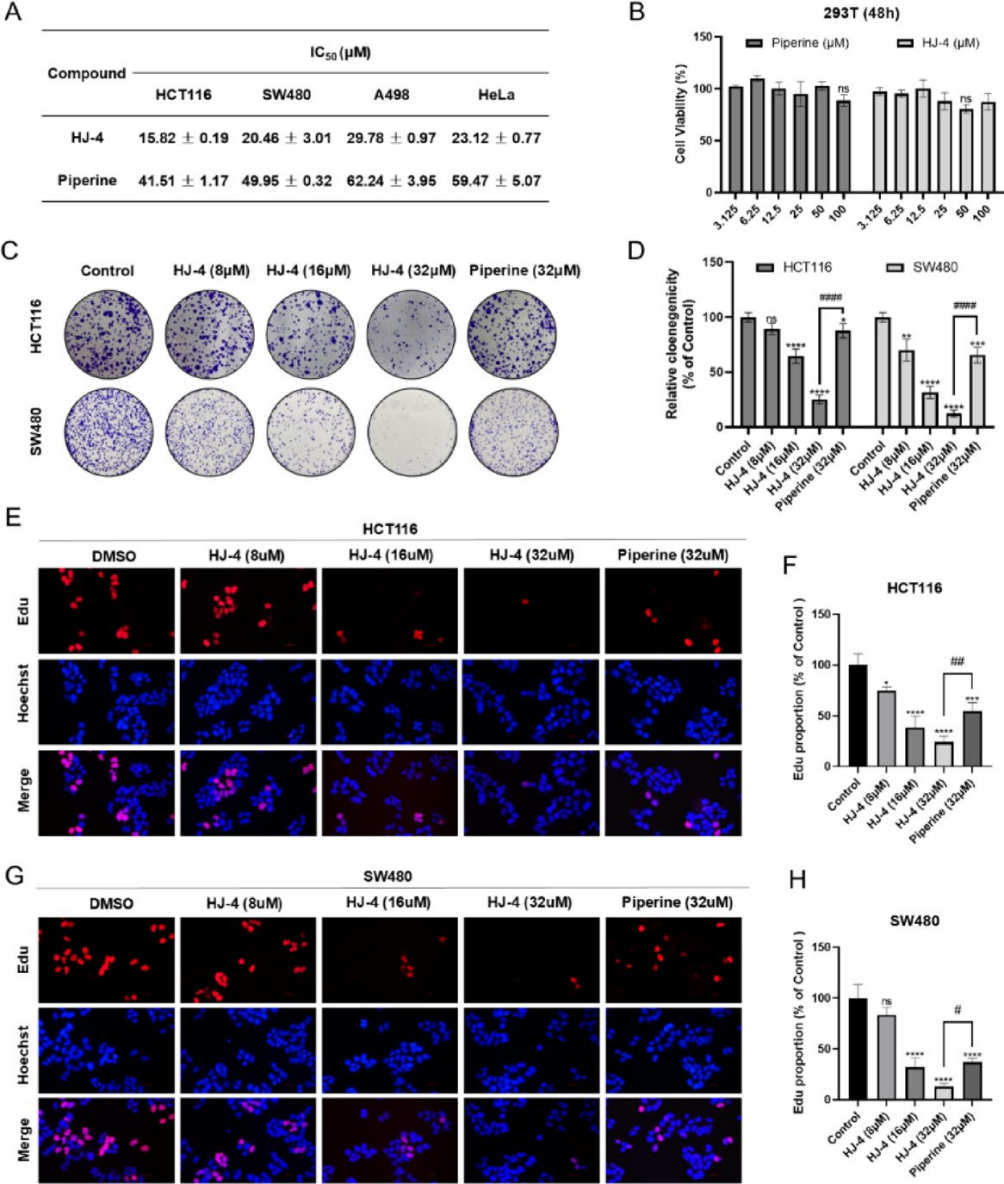
Evaluation of the in vitro antitumor activity of HJ-4. (**A**) IC_50_ values of HJ-4 and piperine in HCT116, SW480, A498, and HeLa cells after 24 h treatment with gradient concentrations. Compared to piperine, HJ-4 exhibited significantly enhanced efficacy, particularly in HCT116 cells (HJ-4: IC_50_ = 15.82 ± 0.19 µM; piperine: IC_50_ = 41.51 ± 1.17 µM). (**B**) Cell viability of 293T cells treated with HJ-4 or piperine (3.125–100 µM) for 24 h. Both compounds showed comparable cytotoxicity in 293T cells, highlighting the tumor selectivity of HJ-4. (**C**–**D**) Colony formation assays in HCT116 and SW480 cells treated with HJ-4 (8–32 µM) or piperine (32 µM). Representative images (**C**) and quantification (**D**) demonstrate a marked reduction in colony formation upon HJ-4 treatment compared to piperine or untreated controls. (**E**,** G**) EdU incorporation assays in HCT116 (**E**) and SW480 (**G**) cells treated with HJ-4 (8–32 µM) or piperine (32 µM). (**F**,** H**) Representative images show decreased EdU-positive cells following HJ-4 treatment, consistent with inhibition of DNA synthesis. Scale bar = 100 μm. Quantitative analysis confirmed statistically significant differences. Data are presented as mean ± SD (*n* = 3). *p < 0.05, **p < 0.01, ***p < 0.001, ****p < 0.0001; #*p* < 0.05, ##*p* < 0.01, ###*p* < 0.001, ####*p* < 0.0001.

Human renal epithelial cells (293T) were used as a non-tumorigenic control to evaluate the in vitro antitumor activity of HJ-4 across a panel of cancer cell lines, including renal (A498), cervical (HeLa), and colorectal (HCT116 and SW480) cancer cells. Cell viability was assessed using the MTT assay, and results showed that HJ-4 significantly inhibited the growth of these cancer cells. Notably, HJ-4 exhibited a markedly lower IC_50_ value in HCT116 cells compared to piperine (HJ-4: IC_50_ = 15.82 ± 0.19 µM; piperine: IC_50_ = 41.51 ± 1.17 µM), demonstrating superior efficacy (Fig. 2A). To evaluate tumor selectivity, the cytotoxicity of HJ-4 was compared in normal 293T cells. The results indicated that HJ-4 exhibited minimal cytotoxicity in 293T cells, comparable to that observed with piperine (Fig. 2B). This suggests that HJ-4 preferentially targets tumor cells while sparing normal cells. Based on these findings and preliminary dose-response experiments, 8 µM was selected as the starting concentration for subsequent cellular assays. This concentration produced detectable biological effects in both colorectal cancer cell lines, making it suitable for mechanistic studies. The antiproliferative effect of HJ-4 was further examined using a colony formation assay. Compared with untreated controls and piperine-treated cells, HJ-4 significantly reduced both the number and size of colonies formed by HCT116 and SW480 cells (Fig. 2C and D). Additionally, EdU incorporation assays were performed to evaluate the effect of HJ-4 on DNA synthesis. HJ-4 treatment led to a concentration-dependent reduction in EdU-positive cells, indicating its ability to inhibit DNA replication (Fig. 2E and G). Quantitative analysis confirmed that HJ-4 exerted significantly stronger inhibitory effects than piperine (Fig. 2F and H). Collectively, these findings identify HJ-4 as a potent inhibitor of tumor cell proliferation with high tumor selectivity, making it a promising candidate for further mechanistic studies.

### HJ-4 inhibits adhesion, migration and invasion of colon cancer cells

**Fig. 3:**
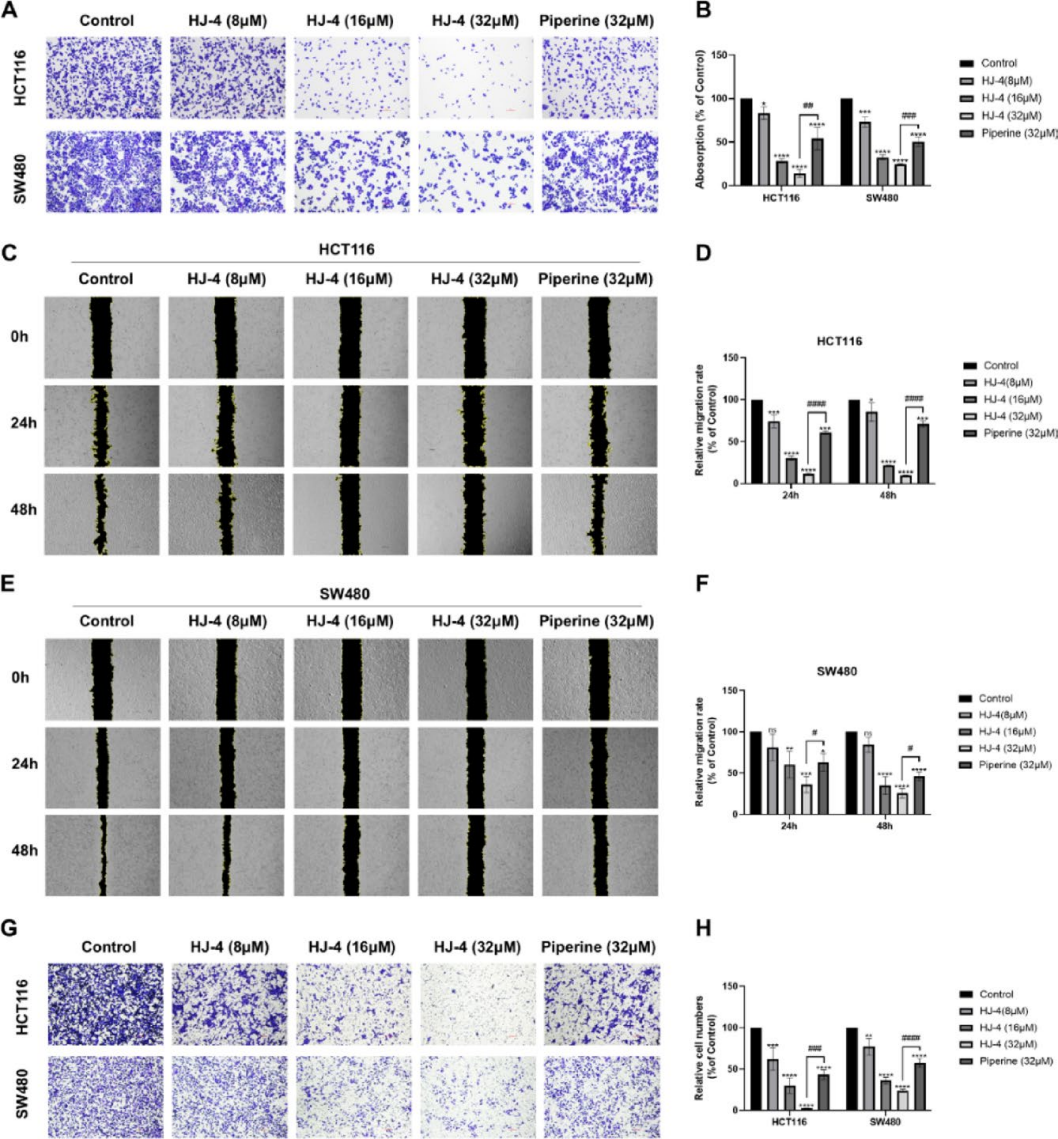
Effects of HJ-4 on adhesion, migration and invasion of HCT116 and SW480 cells. (**A**–**B**) Cell adhesion assay. (**A**) Representative images showed dose-dependent inhibition of cell adhesion by HJ-4 (8 µM, 16 µM, and 32 µM) compared to piperine (32 µM). (**B**) Quantitative analysis of cell adhesion. (**C**–**F**) Scratch-wound healing assay. (**C**,** E**) Representative images of wound healing in HCT116 (**C**) and SW480 (**E**) cells treated with HJ-4 (8–32 µM) or piperine (32 µM) at 0, 24, and 48 h. (**D**,** F**) Quantitative analysis showed that HJ-4 exhibited dose-dependent migration inhibition, which far exceeded the effect of piperine. (**G**–**H**) Transwell invasion assay. (**G**) Representative images show reduced invasion of HCT116 and SW480 cells treated with HJ-4 (8–32 µM) or piperine (32 µM). (**H**) Quantitative analysis demonstrates that HJ-4 at 8 µM achieves invasion inhibition comparable to 32 µM piperine and exhibits greater efficacy at higher concentrations. Data are presented as mean ± SD (*n* = 3). *p < 0.05, **p < 0.01, ***p < 0.001, ****p < 0.0001; #*p* < 0.05, ##*p* < 0.01, ###*p* < 0.001, ####*p* < 0.0001.

Adhesion, migration, and invasion assays were performed in colon cancer cell lines HCT116 and SW480 to evaluate the anti-metastatic effects of HJ-4. In the cell adhesion assays, HJ-4 significantly inhibited the adhesion of both HCT116 and SW480 cells in a dose-dependent manner. As shown in Fig. 3A, higher concentrations of HJ-4 (16 µM and 32 µM) exhibited markedly stronger inhibitory effects than piperine. Quantitative analysis confirmed these results, demonstrating significant adhesion inhibition by HJ-4 at all tested concentrations (Fig. 3B). These findings were further supported by scratch wound healing assays. In HCT116 cells, HJ-4 inhibited cell migration in a dose-dependent fashion, with significantly greater efficacy than piperine (Fig. 3C and D). Similar results were observed in SW480 cells (Fig. 3E and F). Additionally, transwell invasion assays revealed a potent anti-invasion effect of HJ-4. At 8 µM, HJ-4 significantly reduced the number of invading cells, with activity comparable to that of 32 µM piperine. At higher concentrations, HJ-4 exhibited significantly greater inhibition of invasion than piperine (Fig. 3G and H). Collectively, these results indicate that HJ-4 is a potent inhibitor of colon cancer cell adhesion, migration, and invasion, with superior efficacy compared to piperine, underscoring its potential as a promising anti-metastatic agent.

### HJ-4 potently inhibits angiogenesis and tumor growth in colon cancer models

To evaluate the anti-angiogenic and anti-tumor activities of HJ-4, we employed the chick embryo chorioallantoic membrane (CAM) model based on the following considerations: (1) The CAM model is simple to operate, time-efficient, cost-effective, and subject to minimal ethical restrictions, making it suitable for the preliminary screening of novel compounds; (2) its rich vascular network allows for intuitive observation of tumor growth and angiogenesis; (3) our laboratory has established a well-optimized CAM assay system with strong technical feasibility.

**Fig. 4:**
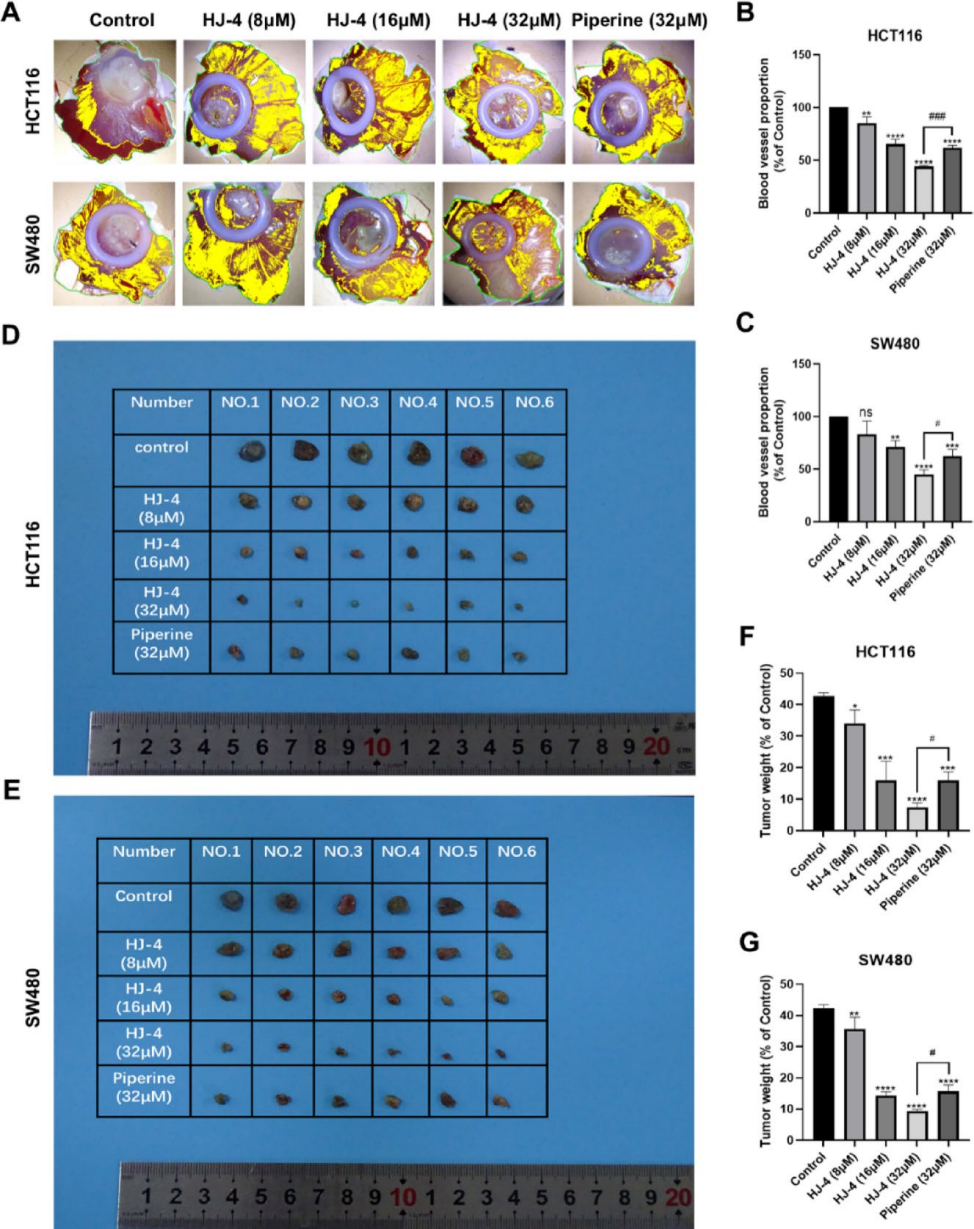
HJ-4 suppresses angiogenesis and tumor growth in colon cancer models. Colon cancer spheroids derived from HCT116 and SW480 cells were implanted into the vascular-rich region of the CAM. HJ-4 (8, 16, and 32 µM) or the positive control Piperine (32 µM) was topically applied to the tumor area daily for 7 consecutive days. Tumors were then excised, weighed, and vascular-like structures were recorded. (**A**) Representative images of vascular-like structures formed after treatment with HJ-4 (8, 16, 32 µM) or Piperine (32 µM). (**B**–**C**) Quantitative analysis of vascular-like structure formation in HCT116 (**B**) and SW480 (**C**) cells. HJ-4 significantly inhibited angiogenesis in a dose-dependent manner compared to the control group. Data are presented as mean ± SD (*n* = 3). (**D**–**E**) Representative images of xenograft tumors derived from HCT116 (**D**) and SW480 (**E**) cells treated with HJ-4 or Piperine. (**F**–**G**) Quantitative analysis of tumor weights in HCT116 (**F**) and SW480 (**G**) xenograft models. HJ-4 significantly reduced tumor weight in a dose-dependent manner and demonstrated greater efficacy than Piperine at 32 µM. Data are presented as mean ± SD (*n* = 6). *p < 0.05, **p < 0.01, ***p < 0.001, ****p < 0.0001; #*p* < 0.05, ##*p* < 0.01, ###*p* < 0.001, ####*p* < 0.0001.

Representative images (Fig. 4A) show that HJ-4 significantly inhibited the formation of vascular-like structures in a dose-dependent manner. As the concentration increased, the construction of the vascular network was progressively disrupted. Quantitative analysis further confirmed that HJ-4 exhibited markedly stronger anti-angiogenic effects than piperine. At higher concentrations, HJ-4 suppressed angiogenesis induced by both cell lines to less than 50% of the control levels (Fig. 4B and C), indicating its potent inhibitory effect on tumor-driven neovascularization. In the CAM xenograft model, representative images (Fig. 4D and F) demonstrate that HJ-4 significantly suppressed tumor growth. Quantification of tumor weight (Fig. 4E and G) revealed that HJ-4 inhibited tumor volume in a dose-dependent manner and was effective across all tested concentrations. Notably, at 32 µM, tumor weight was reduced by more than 70% compared to the control group, with superior efficacy to piperine at the same concentration. HJ-4 simultaneously inhibited tumor growth and angiogenesis, as evidenced by the significant reduction in vessel branching and vascular density observed in the CAM model, highlighting its potential for synergistic anticancer effects. Taken together, these results demonstrate the potent anti-angiogenic and anti-tumor properties of HJ-4, supporting its potential as a promising candidate for colorectal cancer therapy.

### HJ-4 induces apoptosis via activation of p53 signaling in colon cancer cells

**Fig. 5:**
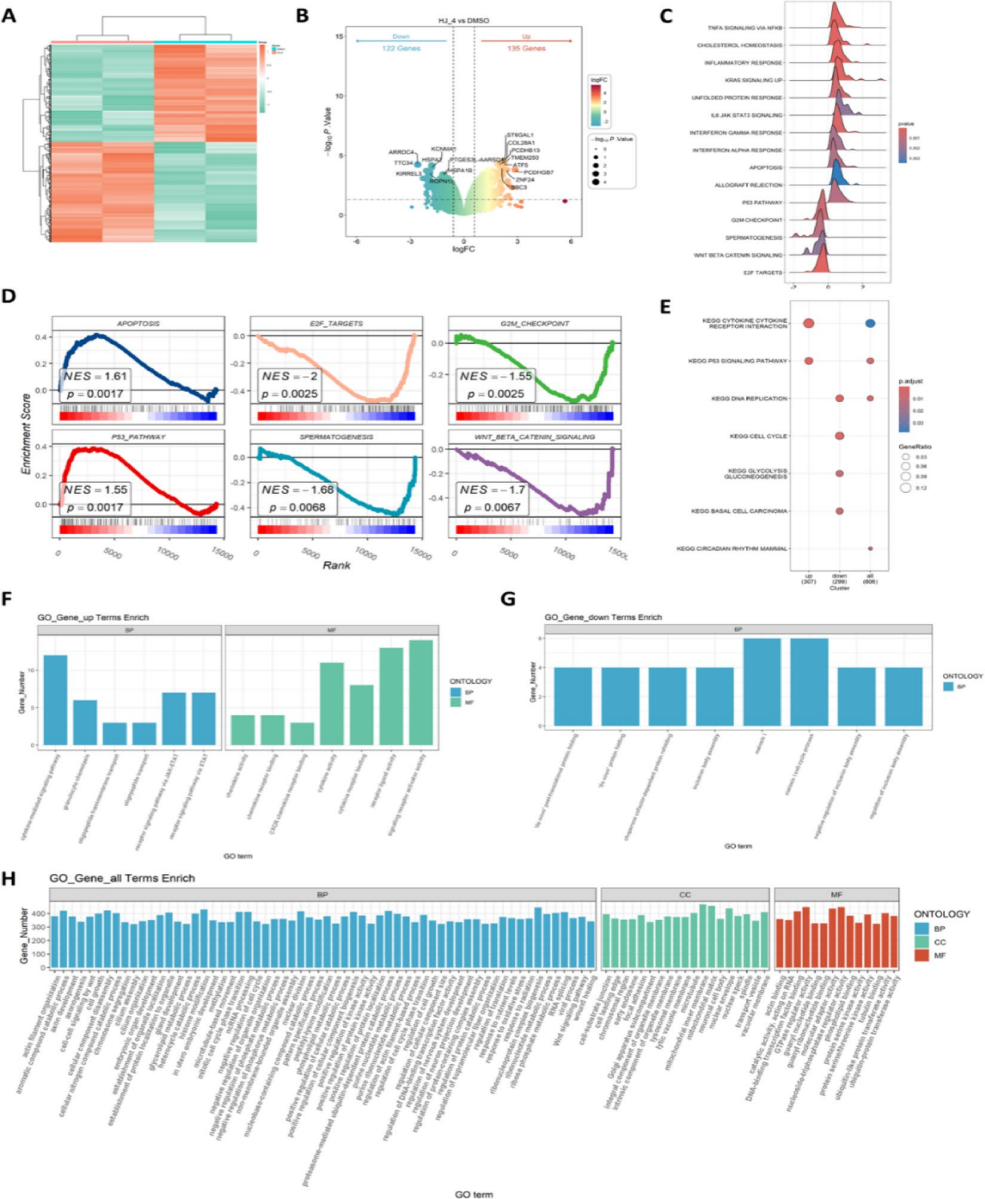
Transcriptomic analysis of HCT116 cells treated with HJ-4, emphasizing the activation of p53 signaling and apoptosis pathways. (**A**–**B**) Heat map (**A**) and volcano map (**B**) of differentially expressed genes in HCT116 cells treated with HJ-4 (16 µM). A total of 257 differentially expressed genes were screened compared with the control group, among which pro-apoptotic genes (e.g., PUMA, also known as BBC3) were significantly up-regulated (*p* < 0.05, |log2-fold change| > 1.2). (**C**) Ridge plot reveals the enrichment of p53 signaling, apoptosis and cell cycle regulation pathways. (**D**) GSEA plots. Positive enrichment of p53 signaling (NES = 1.55, *p* = 0.0017) and apoptosis pathways (NES = 1.61, *p* = 0.0017) indicates activation of tumor suppressive mechanisms by HJ-4. Negative enrichment of E2F targets (NES = -2.00, *p* = 0.0025) and Wnt/β-catenin signaling (NES = -1.70, *p* = 0.0067) reveals inhibition of oncogenic pathways. (**E**) KEGG pathway analysis of differentially expressed genes. Significant pathways include p53 signaling, DNA replication, and cell cycle regulation, underscoring HJ-4’s dual role in promoting apoptosis and suppressing proliferation. (**F**–**G**) GO enrichment analysis of up-regulated (**F**) and down-regulated (**G**) genes. Up-regulated genes were associated with apoptosis and stress response, while the down-regulated genes were involved in mitotic spindle organization and chromatin regulation. (**H**) Comprehensive GO analysis of all differentially expressed genes in the ontology, including biological process (BP), molecular function (MF), and cell component (CC). The bar chart highlights the regulation of pathways involved in apoptosis and nuclear tissue.

To elucidate the molecular mechanisms underlying the antitumor effects of HJ-4, transcriptomic and protein expression analyses were performed. Transcriptome analysis identified a total of 257 differentially expressed genes, among which pro-apoptotic genes—such as PUMA (also known as BBC3)—were significantly upregulated (Fig. 5A and B). Gene Set Enrichment Analysis (GSEA) revealed that HJ-4 markedly activated the p53 signaling pathway (NES = 1.55, *p* = 0.0017) and the apoptosis pathway (NES = 1.61, *p* = 0.0017), while simultaneously inhibiting multiple oncogenic pathways, including E2F target genes (NES = - 2.00, *p* = 0.0025) and Wnt/β-catenin signaling (NES = -1.70, *p* = 0.0067) (Fig. 5C and D). Pathway enrichment analysis further confirmed sustained activation of the p53 pathway in the HJ-4-treated group (Fig. 5E), underscoring its central role in cell fate regulation. Additionally, genes associated with DNA replication—such as GDF15, BBC3(PUMA), SESN2, TERF2IP, and ZNF24—were significantly upregulated, suggesting that HJ-4 may exert its antitumor effects in part through a p53-mediated DNA damage response. Moreover, Gene Ontology (GO) enrichment analysis comprehensively characterized the cellular activity changes following HJ-4 treatment across three dimensions: biological processes (BP), molecular functions (MF), and cellular components (CC). In addition to classical apoptosis pathways, processes such as cellular stress response, chromatin remodeling, and nuclear chromatin binding were significantly enriched (Fig. 5F and H), further supporting the multifaceted role of HJ-4 in regulating cellular behavior.

**Fig. 6:**
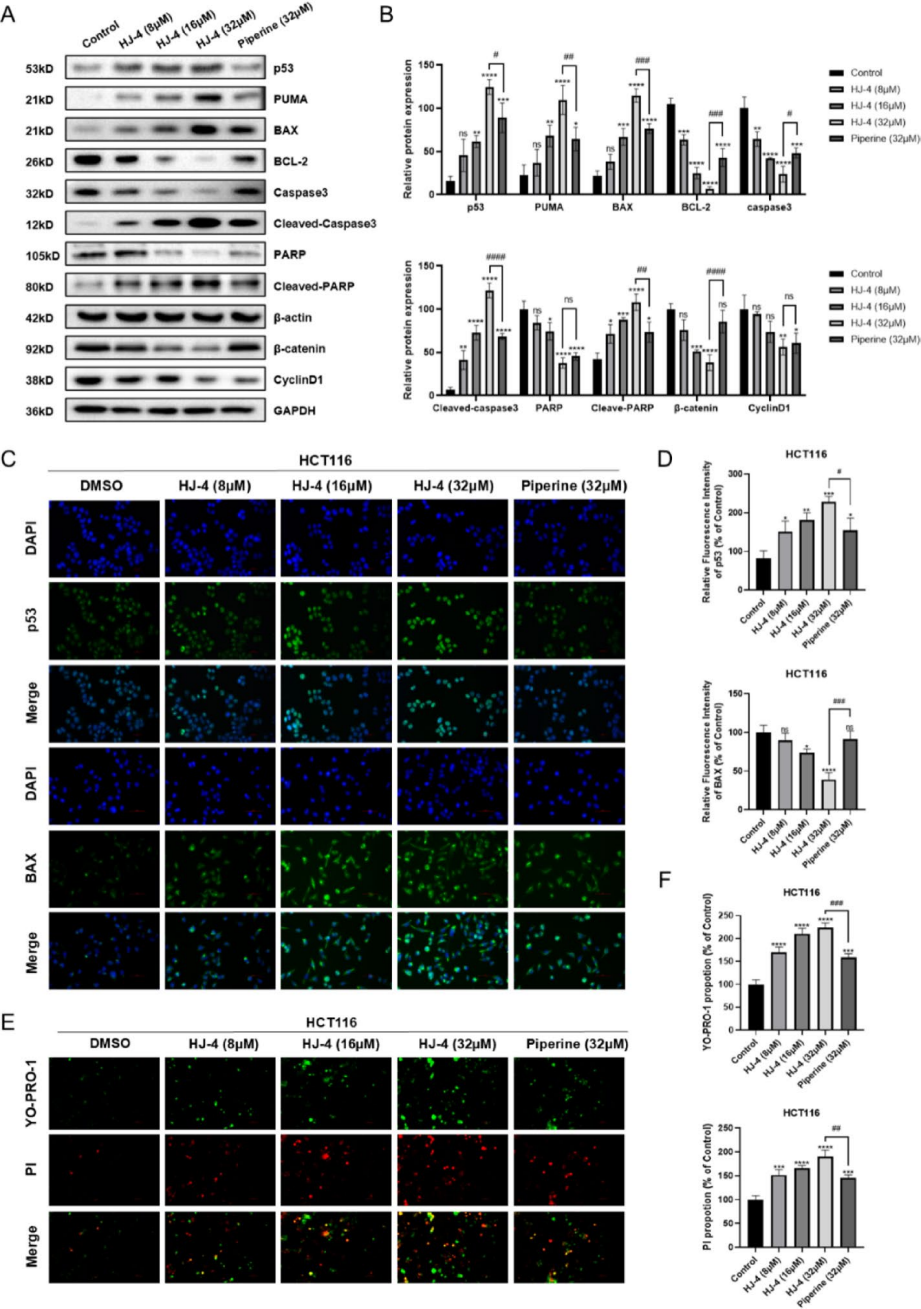
HJ-4 induces apoptosis through p53 pathway activation in HCT116 cells. (**A**) Western blot analysis of apoptosis-related proteins (p53, PUMA, BAX, BCL-2, cleaved caspase-3, and cleaved PARP) and Wnt/β-catenin signaling proteins (β-catenin and Cyclin D1) in cells treated with HJ-4 (8 µM, 16 µM, and 32 µM) or piperine (32 µM). β-actin was used as a loading control. (**B**) Quantification of protein expression changes. HJ-4 induced a dose-dependent upregulation of pro-apoptotic markers and downregulation of BCL-2, β-catenin, and Cyclin D1. (**C**–**D**) Immunofluorescence analysis of p53 and BAX expression. Nuclei were stained with DAPI. Representative images show increased expression of p53 and BAX following HJ-4 treatment. At 32 µM, HJ-4 exhibited a notably stronger effect than piperine. (**E**–**F**) Live-cell staining using YO-PRO-1 (green, early apoptosis) and PI (red, late apoptosis). Merged images reveal dose-dependent apoptosis induced by HJ-4. Scale bar = 100 μm. Data are presented as mean ± SD (*n* = 3). *p < 0.05, **p < 0.01, ***p < 0.001, ****p < 0.0001; #*p* < 0.05, ##*p* < 0.01, ###*p* < 0.001, ####*p* < 0.0001.

At the protein level, Western blot analysis confirmed a dose-dependent upregulation of p53, PUMA, and BAX, along with the activation of cleaved caspase-3 and cleaved PARP (Fig. 6A). Meanwhile, HJ-4 significantly downregulated the expression of the anti-apoptotic protein BCL-2, as well as β-catenin—a key marker of the Wnt/β-catenin signaling pathway—and its downstream target, Cyclin D1 (Figs. S5 and S6). Quantitative analysis (Fig. 6B) further demonstrated that increasing concentrations of HJ-4 led to a progressive activation of apoptotic markers and suppression of survival pathways. Immunofluorescence analysis additionally confirmed the upregulation of p53 and BAX in HJ-4-treated cells (Fig. 6C). Notably, 8 µM HJ-4 induced a level of p53 activation comparable to that of 32 µM piperine, while higher concentrations (16 µM and 32 µM) elicited markedly stronger effects (Fig. 6D). To monitor the apoptotic process in real time, live-cell staining with YO-PRO-1 and PI was performed. HJ-4 induced a dose-dependent increase in YO-PRO-1 (green, early apoptosis) and PI (red, late apoptosis) fluorescence signals, indicating its potent ability to trigger apoptotic cell death (Fig. 6E). Quantitative analysis confirmed that, at equivalent concentrations, HJ-4 exhibited significantly greater pro-apoptotic activity than piperine (Fig. 6F). These findings suggest that HJ-4 exerts its anti-tumor effects by activating the p53-mediated apoptotic pathway and inhibiting oncogenic signaling, highlighting its potential as a targeted therapeutic agent for colorectal cancer.

## Discussion

This study systematically evaluated the antitumor activity and mechanism of action of HJ-4, a novel piperine derivative in colorectal cancer. The results showed that HJ-4 exhibited good tumor selectivity and multi-target intervention ability, significantly inhibiting the proliferation, migration, invasion, angiogenesis, and growth of colorectal cancer cells, demonstrating a high potential for clinical translation.

HJ-4 exhibits excellent target specificity, demonstrating significantly stronger cytotoxicity than its parent compound, piperine, in the HCT116 cell model, with a 2.6-fold increase in potency. Notably, while HJ-4 effectively eliminates cancer cells, it shows minimal toxicity toward normal human renal epithelial cells, indicating a favorable safety profile. In addition to its selective cytotoxicity, HJ-4 displays multi-faceted anti-tumor activities. In vitro studies revealed that it not only markedly inhibits the proliferation of colorectal cancer cells but also effectively suppresses their adhesion, migration, and invasion, highlighting its potent anti-proliferative and anti-invasive properties^19^. More importantly, in vivo experiments have demonstrated that HJ-4 exerts a dual inhibitory effect by suppressing angiogenesis within tumor tissues and significantly blocking tumor growth^20^. This mechanism simultaneously targets both the tumor and its microenvironment, aligning with current anti-angiogenic strategies and offering promising potential for synergistic therapeutic outcomes.

In mechanistic studies, transcriptomic analysis revealed that HJ-4 simultaneously regulates both p53-dependent and independent pathways, including the inhibition of E2F_TARGETS and Wnt/β-catenin signaling pathways^21–23^. Further molecular biology experiments demonstrated that HJ-4 exerts its core antitumor activity by activating the p53-mediated apoptotic pathway^24^ characterized by the upregulation of pro-apoptotic proteins such as PUMA^25^ and BAX^26^ and the downregulation of anti-apoptotic factors like BCL-2^27^, ultimately leading to the activation of the caspase cascade. In parallel, the observed inhibition of cell proliferation and the downregulation of β-catenin and Cyclin D1 further support the inhibitory effect of HJ-4 on the Wnt/β-catenin signaling pathway. This multi-targeted mechanism provides a promising strategy to overcome tumor heterogeneity.

The clarity of the molecular mechanisms of HJ-4, along with its significant antitumor activity both in vitro and in vivo, lays a solid foundation for its preclinical translation and widespread application in malignant tumors such as colorectal cancer. However, several key issues still need to be explored, including elucidating its pharmacokinetic properties in vivo, establishing a safe dose range through preclinical toxicity studies, expanding its efficacy evaluation in p53 wild-type and mutant tumors, and exploring combination therapy^28^ strategies with existing treatments.

In summary, HJ-4, as an anticancer candidate drug derived from the structural optimization of piperine, shows significant advantages in multi-target antitumor effects and represents an important advance in the development of drugs for colorectal cancer. This study systematically elucidated its mechanism of action and in vitro and in vivo activity, laying a solid foundation for its preclinical research and future application in solid tumors such as colorectal cancer.

## Supplementary Information

Below is the link to the electronic supplementary material.

Supplementary Material 1

## Data Availability

The data discussed in this article have been deposited in the Gene Expression Omnibus (GEO) database at NCBI (Edgar et al., 2002) and are accessible through GEO Series Accession Number GSE282967 (https://www.ncbi.nlm.nih.gov/geo/query/acc.cgi? acc=GSE282967).
