# Supplementary material for: HJ-4, a novel piperine derivative, inhibits tumor growth and angiogenesis via p53 activation and oncogenic pathway inhibition in colorectal cancer models
Source: Sci Rep. 2025 Sep 29;15:33541. doi: 10.1038/s41598-025-18290-6 (PMC12480698; doi:10.1038/s41598-025-18290-6)
Supplement: Supplementary file 1 — Supplementary Material 1 [file 41598_2025_18290_MOESM1_ESM.docx]

**Supplementary Information for**

**HJ-4, a Novel Piperine Derivative, Inhibits Tumor Growth and Angiogenesis via p53 Activation and Oncogenic Pathway Inhibition in Colorectal Cancer Models**

Luyao Zhang^1#^, Shunfang Liu^2#^, Dan Wang^1#^, Xingyu Zhang^1#^, Zhongke Hu^1^, Xun Zou ^1^, Xiuming Li^1^, Xiujun Wang^1^, Dandan Xu^1*^, Wei Liu^3*^, Bin Liu^1*^

1. Jiangsu Key Laboratory of Marine Pharmaceutical Compound Screening, College of Pharmacy, Jiangsu Ocean University, Lianyungang, 222005, China.
2. Department of Oncology, Tongji Hospital of Tongji Medical College, Huazhong University of Science and Technology, Jiefang Road 1095, Wuhan, 430030, China.
3. Cancer Center and Department of Pharmacology and Toxicology, Medical College of Wisconsin, Milwaukee, WI 53226, USA.

# These authors contributed equally

* [Corresponding author](javascript:;)s

Dr. Dandan Xu: [2024000070@jou.edu.cn](mailto:2024000070@jou.edu.cn). Jiangsu Key Laboratory of Marine Pharmaceutical Compound Screening, College of Pharmacy, Jiangsu Ocean University, Lianyungang 222005, China.

Dr. Wei Liu: [weiliu@mcw.edu](mailto:weiliu@mcw.edu). Cancer Center and Department of Pharmacology and Toxicology, Medical College of Wisconsin, Milwaukee, WI 53226, USA.

Dr. Bin Liu: [liubin@jou.edu.cn](mailto:liubin@jou.edu.cn). Jiangsu Key Laboratory of Marine Pharmaceutical Compound Screening, College of Pharmacy, Jiangsu Ocean University, Lianyungang 222005, China.

**Contents**

1. Figure S1. ^1^H NMR spectra of HJ-4
2. Figure S2. ^13^C NMR spectra of HJ-4
3. Figure S3. HRMS spectra of HJ-4
4. Figure S4. HPLC spectra of HJ-4
5. Figure S5. Original uncropped image of western blot analysis (β-actin, p53, PUMA, BAX, BCL-2, caspase3)
6. Figure S6. Original uncropped image of western blot analysis (Cleaved-caspase3, PARP, cleaved-PARP, GAPDH, β-catenin, CyclinD1)

**
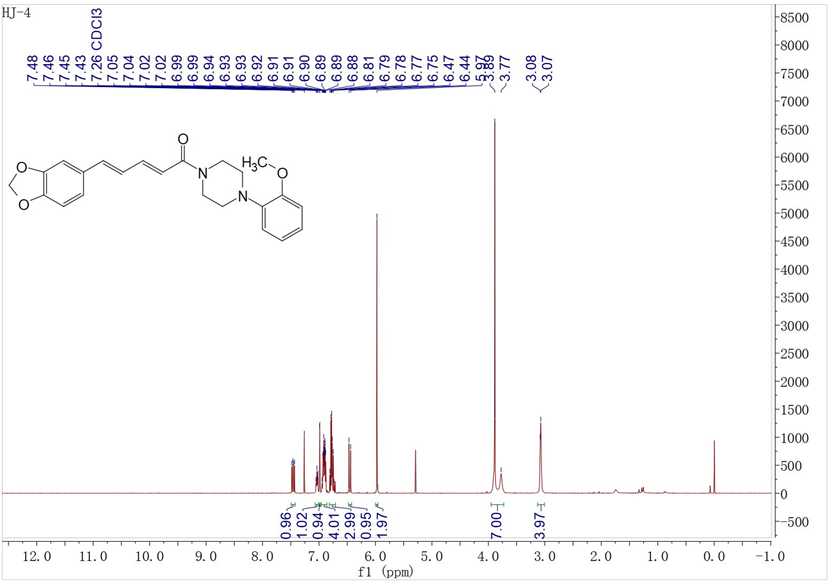
**

Figure S1. ^1^H NMR spectra of HJ-4.

^1^H NMR (500 MHz, CDCl_3_), δ 7.46 (dd, *J* = 14.6, 9.9 Hz, 1H), 7.07–7.00 (m, 1H), 6.99 (d, *J* = 1.6 Hz, 1H), 6.96–6.86 (m, 4H), 6.80–6.71 (m, 3H), 6.45 (d, *J* = 14.6 Hz, 1H), 5.97 (s, 2H), 3.89–3.77 (m, 7H), 3.10–3.04 (m, 4H).

**
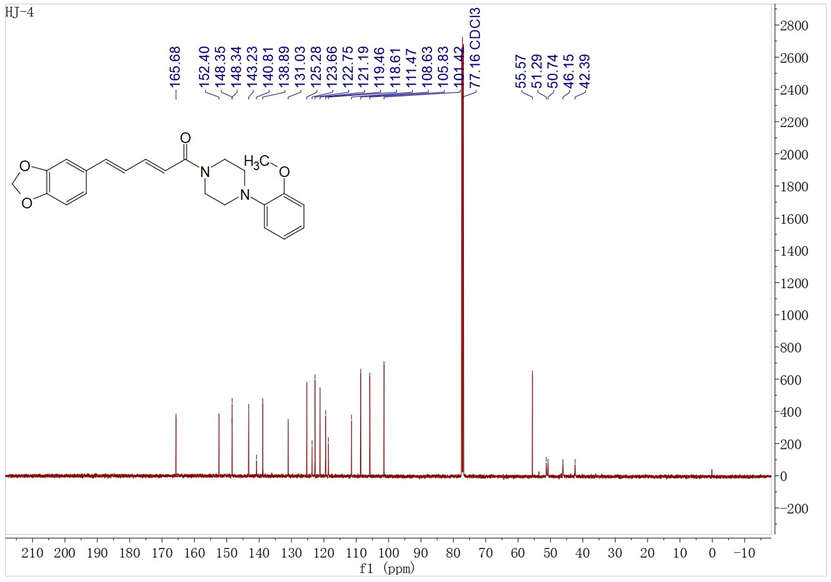
**

Figure S2. ^13^C NMR spectra of HJ-4.

^13^C NMR (126 MHz, CDCl_3_), δ 165.7, 152.4, 148.4, 148.3, 143.2, 140.8, 138.9, 131.0, 125.3, 123.7, 122.7, 121.2, 119.5, 118.6, 111.5, 108.6, 105.8, 101.4, 55.6, 51.3, 50.7, 46.2, 42.4.


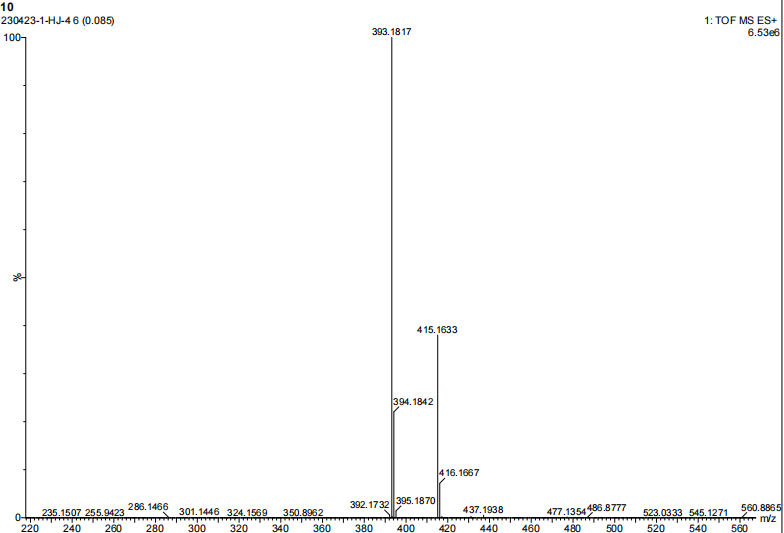


Figure S3. HRMS spectra of HJ-4.

HRMS *m/z*: [M+H]^+^ calcd for C_23_H_25_N_2_O_4_: 393.1809, found 393.1817.


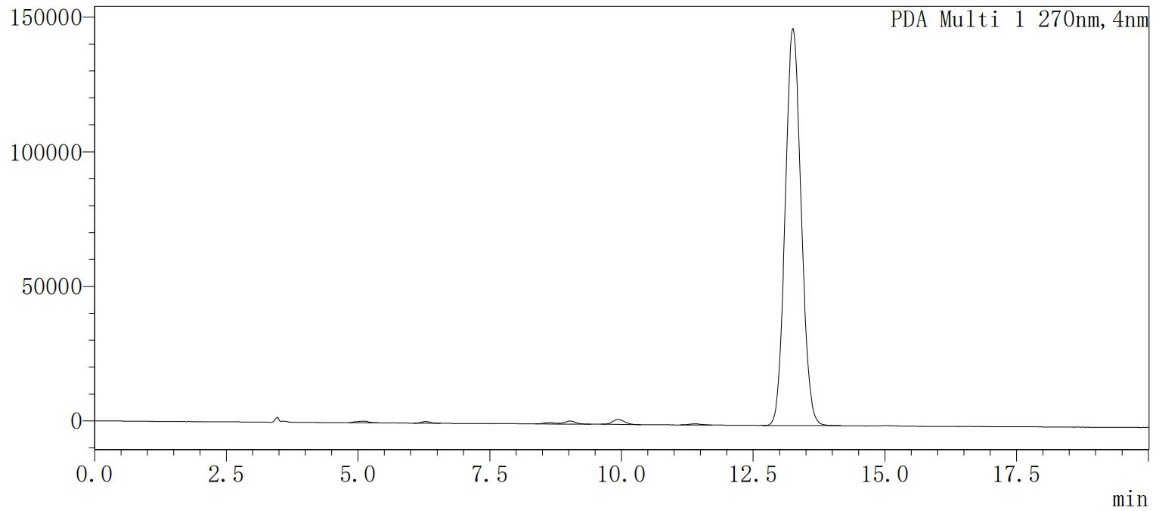


Figure S4. HPLC spectra of HJ-4.

HJ-4 was 97.5% pure analyzed by Shimadzu LC20AD high performance liquid chromatography (HPLC) coupled with a diode array detector (DAD) and the Inertsil ODS-3 column (EC-C18, 4.6 mm × 250 mm, 5 μm) with the eluent 77% CH_3_OH in pure water. The analysis process lasted for 20 min with a flow rate of 1.0 mL/min.


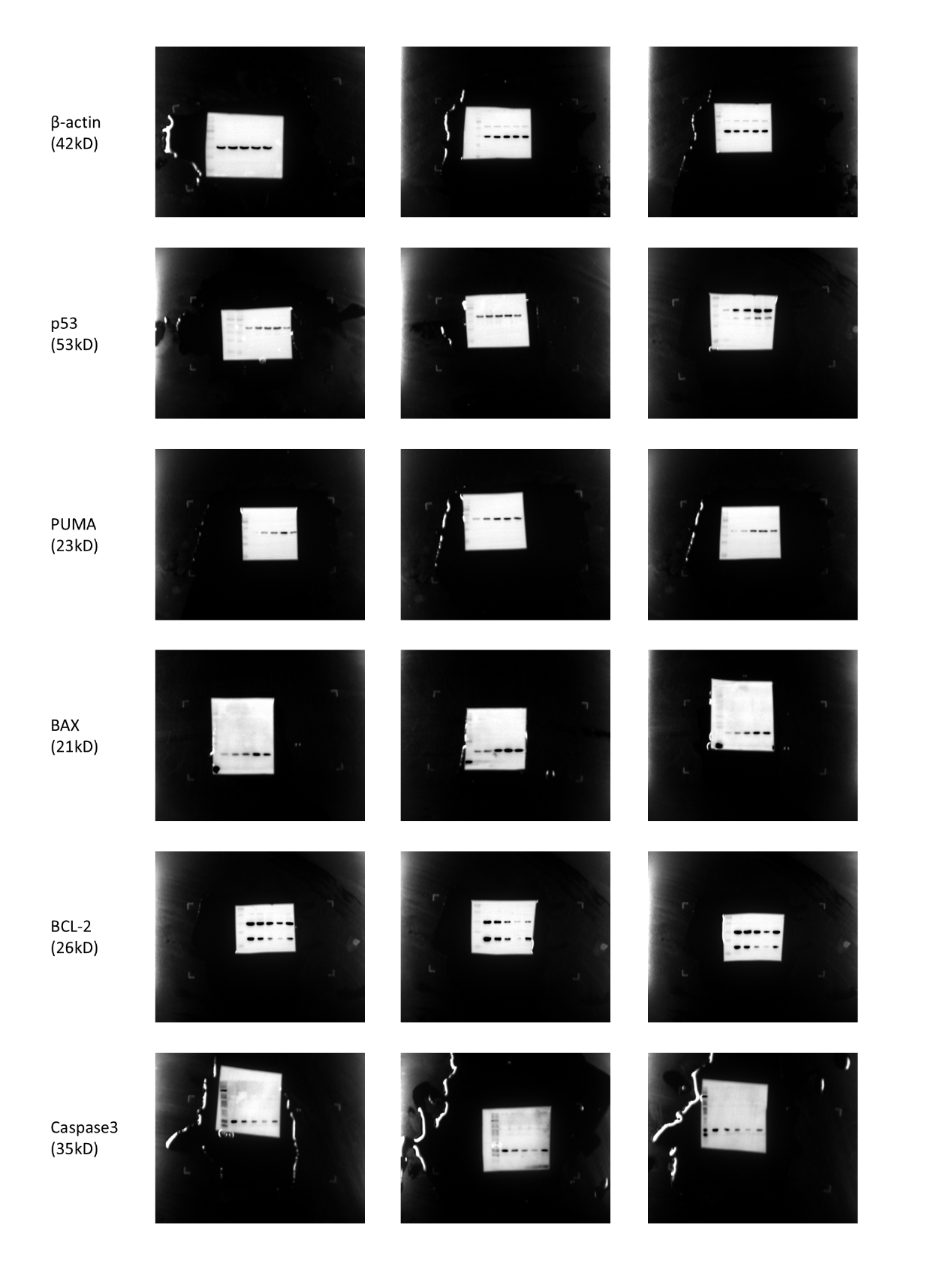


Figure S5. Original uncropped image of western blot analysis (β-actin, p53, PUMA, BAX, BCL-2, caspase3).


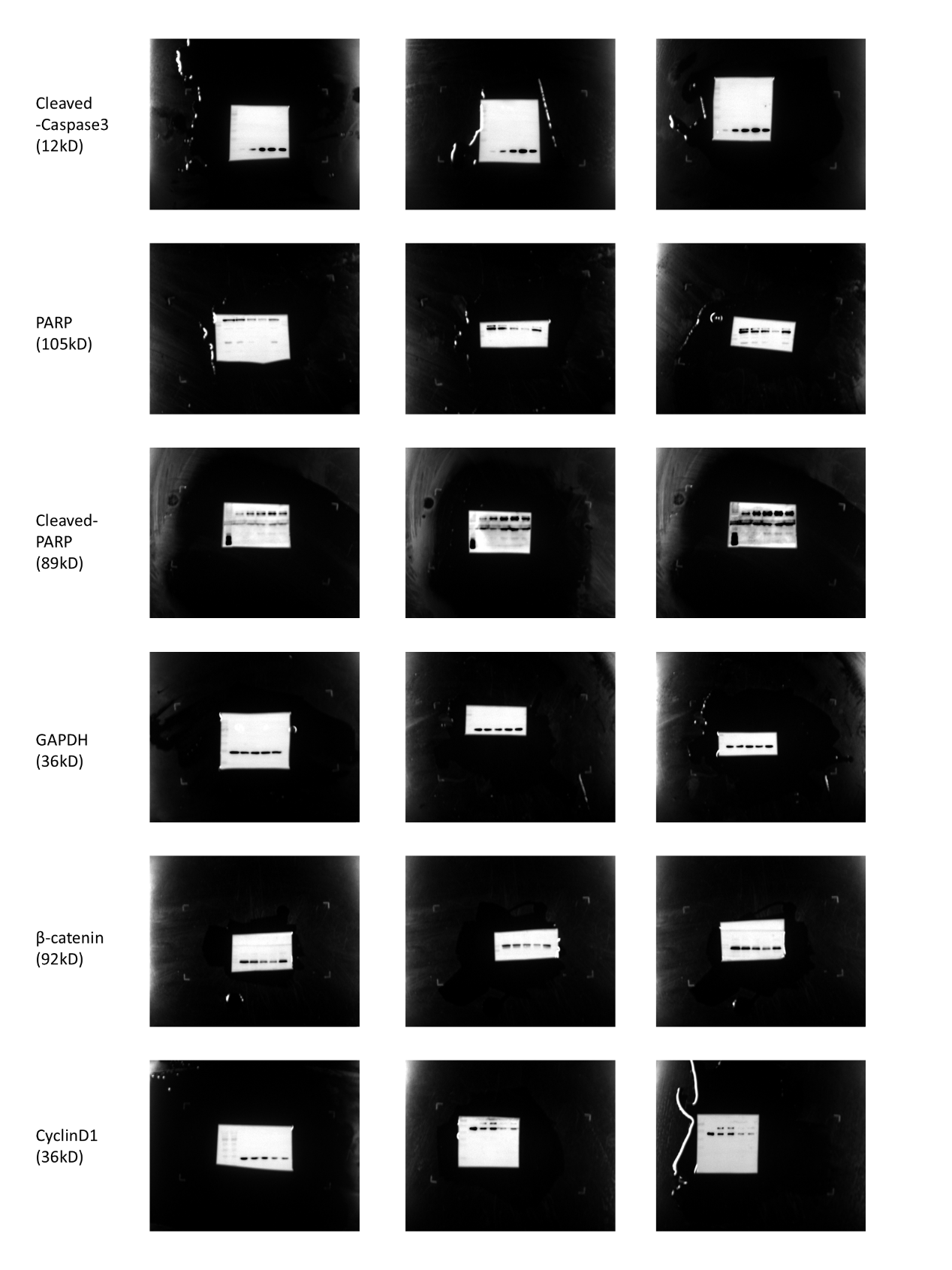


Figure S6. Original uncropped image of western blot analysis (Cleaved-caspase3, PARP, cleaved-PARP, GAPDH, β-catenin, CyclinD1).

.
